# Supplementary material for: Novel Graphene/In2O3 Nanocubes Preparation and Selective Electrochemical Detection for L-Lysine of Camellia nitidissima Chi
Source: Materials (Basel). 2020 Apr 24;13(8):1999. doi: 10.3390/ma13081999 (PMC7215595; doi:10.3390/ma13081999)
Supplement: Supplementary file 1 [file materials-13-01999-s001.pdf]

materials

Supplementary Materials

Novel Graphene/In_2O_3 Nanocubes Preparation and Selective Electrochemical Detection for L-Lysine of *Camellia nitidissima* Chi

Jinsheng Cheng [1,*,†], Sheng Zhong [2,†], Weihong Wan [1,3], Xiaoyuan Chen [1], Ali Chen [4] and Ying Cheng [3]

1 Henry-Fork School of Food Sciences, Shaoguan University, Shaoguan 512005, China; weihongsgu@163.com (W.W.); xychensgu@126.com (X.C.)
2 Shipai Branch, Dongguan Environmental Protection Bureau, Dongguan 523330, China; jasonwow@163.com
3 Foshan Qionglu Health Tech. Ltd., Foshan 528000, China; yingchenggd@163.com
4 School of Pharmacy, Guangdong Pharmaceutical University, Guangzhou 510006, China; chenali2004@163.com
* Correspondence: chengjins@gmail.com
† These authors contributed equally to this work.

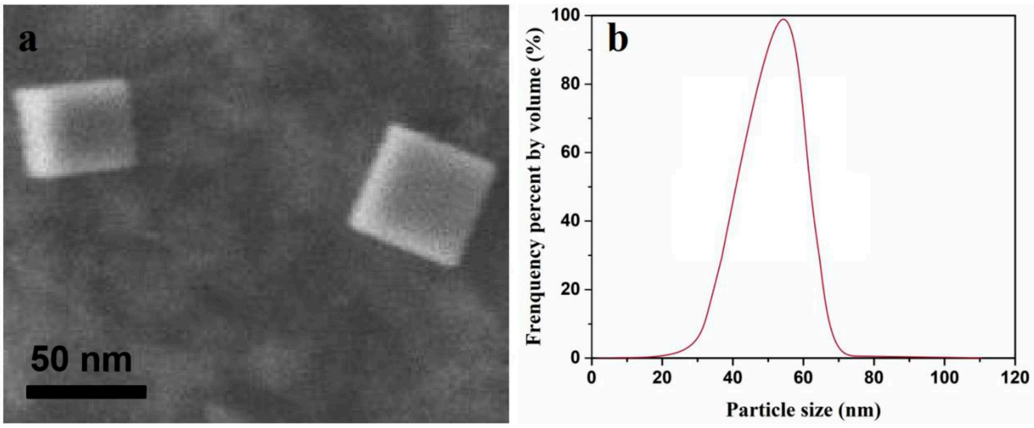

Figure S1. (**a**) SEM images of GR/In_2O_3 nanocubes, (**b**) Particles size distribution image of the prepared GR/In_2O_3 nanocubes.

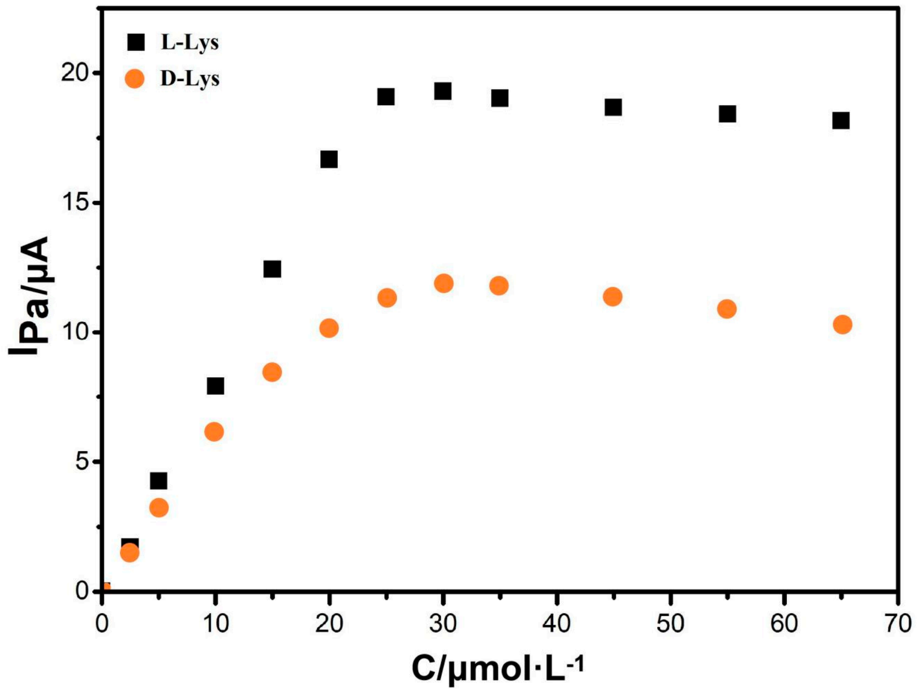

Figure S2. Response curves for the currents of L-Lys and D-Lys by the prepared GR/In_2O_3 nanocubes based electrochemical sensor.

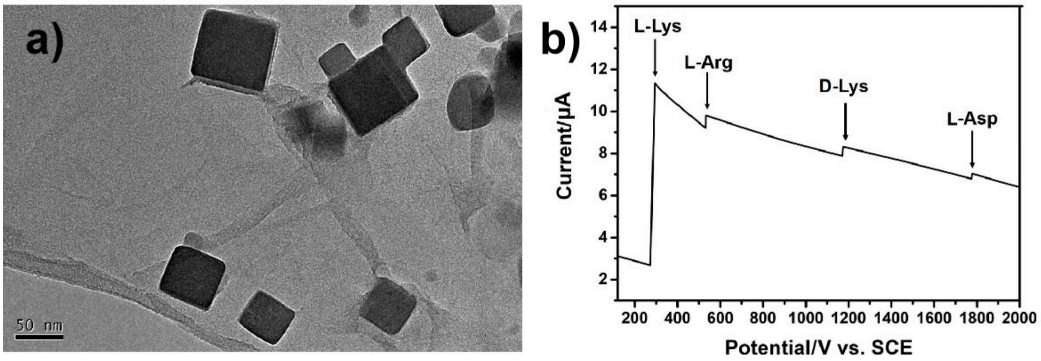

Figure S3. (**a**) Non-uniformity size distributed GR/In_2O_3 nanocubes; (**b**) Current responses obtained at GR/In_2O_3 nanocubes (with non-uniformity size range of 20–100 nm) based electrochemical sensor of amino acids extraction in Camellia Nitidissima Chi (extra addition D-Lys with a concentration of 0.45%), applied potential: 0.85 V.

© 2020 by the authors. Submitted for possible open access publication under the terms and conditions of the Creative Commons Attribution (CC BY) license (http://creativecommons.org/licenses/by/4.0/).
